# Prostate Cancer Screening and Strategies, Florida Behavioral Risk Factor Surveillance System, 2012–2020

**DOI:** 10.5888/pcd20.230203

**Published:** 2023-11-23

**Authors:** Dottington Fullwood, Justine Gunderson, Brandon Snipe, Isela Villasenor, Emelina Asto-Flores, Shannon Pressey, Randy Hale, Folakemi T. Odedina

**Affiliations:** 1 Mayo Clinic, Jacksonville, Florida

**Figure:**
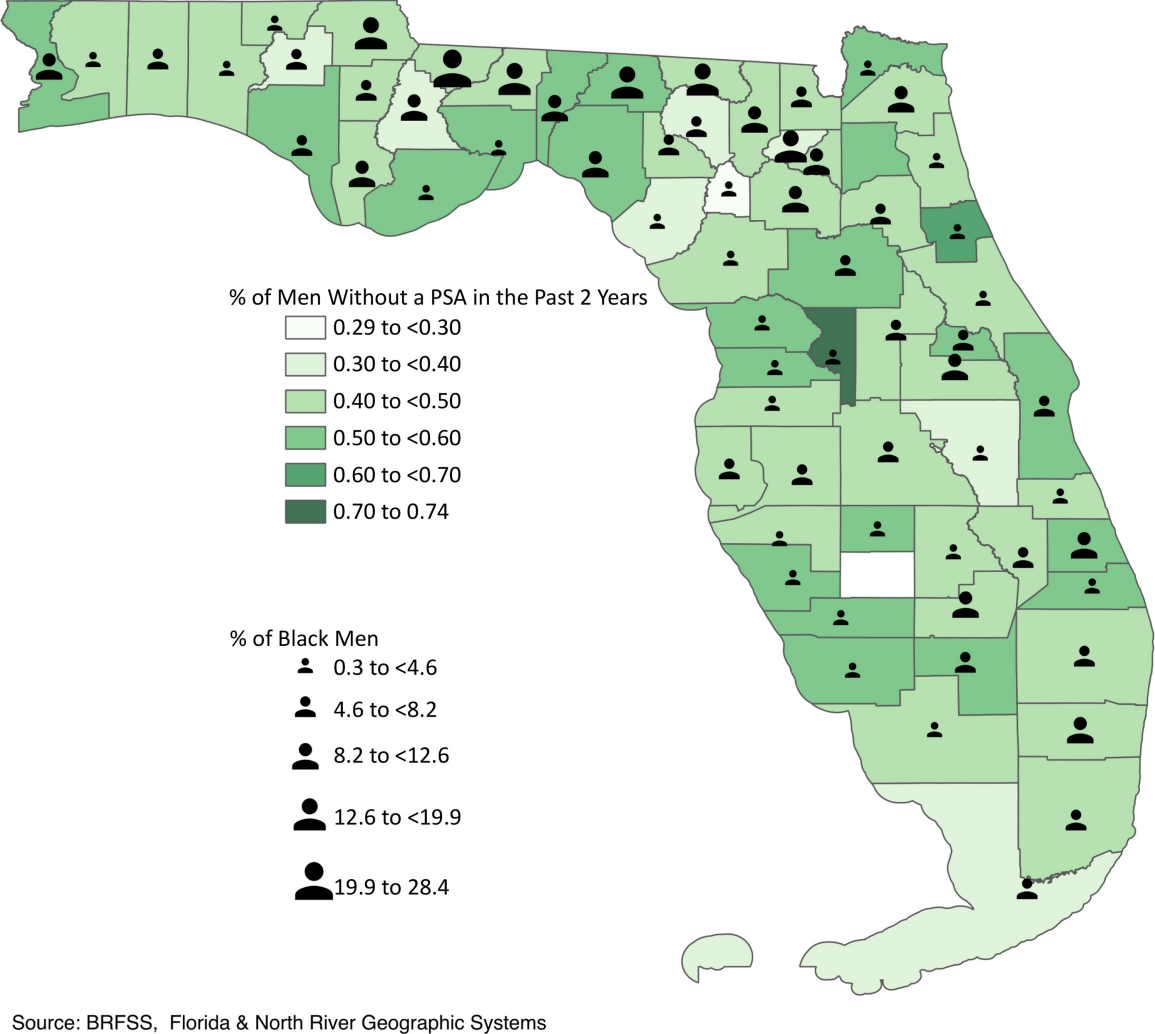
Percentage of men that did not have a prostate-specific antigen (PSA) screening in the past 2 years (1) and percentage of Black men in the overall population across 67 counties in Florida in 2020 (2).

## Background

Recent trends in prostate cancer incidence suggest that widening cancer disparities mean a greater number of prostate cancers in Black men (3). Black men, including US-born and Black immigrants, have the highest prostate cancer incidence and mortality rates of all racial groups (4). Notably, Black men at diagnosis tend to present with a more aggressive or advanced stage prostate cancer (5). This disparity consistently extends to a high proportion of the Black male population and may be driven by a combination of genetic, environmental, social, and economic factors, all of which contribute to widening cancer disparities as a whole (3). However, unresolved conflicts in 2012 concerning prostate-specific antigen (PSA) screening recommendations could explain the disproportionately higher occurrence of negative prostate cancer outcomes due to increases in regional‐stage and distant‐stage diagnoses.

The distribution of prostate cancer across Florida remains uneven, with Black men bearing the greatest burden (6). Although survey research design offers a cost-effective method to collect reliable and valid data among the population, Black respondents with prostate cancer represent a small sample size that is insufficient to provide meaningful statistical analysis and generalizability to the larger population. The Behavioral Risk Factor Surveillance System (BRFSS) represents the best estimation of identifying clusters to improve prostate cancer outcomes despite recruitment strategy limitations.

This study focuses on prostate cancer screening among all reported Black respondents between 2012 and 2020. The geographic unit of analysis was state-level data to identify trends over time in prostate cancer screening. Additionally, we incorporated complete county-level data to show patterns of screening distribution.

## Data and Methods

Sample survey data were obtained from the Florida BRFSS, an annual survey distributed to civilian, noninstitutionalized Florida adults aged 18 years or older, using random-digit–dialed landline and cellular telephone numbers. Florida BRFSS collects information on sociodemographic characteristics, health behaviors, health care access, and preventive health practices, including cancer screening. Prostate cancer screening questions are included on the Florida BRFSS during even-numbered years as part of the rotating core questionnaire (1). This snapshot analysis focuses on trends in PSA testing among Florida men aged 40 years or older between 2012 and 2020. We analyzed the prevalence of respondents who self-reported having their last PSA test within the past 2 years before survey administration for the years 2012, 2014, 2016, 2018, and 2020. Estimates of respondents who reported “don’t know” or “not sure” ranged from 2% to 6% across the 5 waves, resulting in a sample size large enough to permit analyses by race and ethnicity. The analysis included several variables, and certain data were excluded from the study. SAS software version 9.4 (SAS Institute, Inc) was used to calculate weighted prevalence estimates for PSA testing within the past 2 years and to determine the percentage change in PSA testing prevalence over time. The map illustration was analyzed and completed using QGIS version 3.24.3 (Open Source Geospatial Foundation) to show distribution patterns of prostate cancer across Florida.

## Highlights

The percentage of Florida men aged 40 years or older who reported receiving a PSA test in the past 2 years decreased 28.9%, from 51.2% (95% CI, 48.1%–54.4%) in 2012 to 36.4% (95% CI, 33.0%–39.7%) in 2020, representing 503,443 fewer Floridians screened for prostate cancer. Relative to differences in prostate cancer screening prevalence between individual years within the period, the percentage of Florida male residents who received a PSA test in the past 2 years declined from 51.2% in 2012 to 49.7% (95% CI, 47.3%–52.2%) in 2014, a 2.9% decrease; and from 49.7% in 2014 to 46.2% (95% CI, 44.2%–48.2%) in 2016, a 7.0% decrease. A 14.5% reduction occurred between 2016 and 2018, from 46.2% to 39.5% (95% CI, 36.7%–42.4%), and another 7.8% between 2018 and 2020, from 39.5% to 36.4% (95% CI, 33.0%–39.7%).

The investigation of these PSA testing trends by race and ethnicity resulted in substantial differences. In 2012, the highest screening prevalence was for non-Hispanic Black men (54.7%; 95% CI, 43.7%–65.6%) and non-Hispanic White men (53.7%; 95% CI, 50.2%–57.1%), followed by Hispanic men (44.7%; 95% CI, 35.3%–54.1%). The greatest decline in prostate cancer screening between 2012 and 2020 was for non-Hispanic Black men (37.8%), while the screening rate for non-Hispanic White men decreased to 30.4%.

## Action

The continuous increase in prostate cancer incidence from 2014 to 2019 (3) urges immediate action. Our findings indicate a major public health concern, which requires primary prevention strategies to improve informed prostate cancer screening in at-risk Black men. Our work identified specific counties with both a high absence of PSA screening and a greater proportion of Black men, illustrating a significant health disparity (7). The mitigation of geographic hotspots may rely on focused efforts that help decrease the disproportionate burden of prostate cancer in Black men in Florida.

A strength of our study was the use of a large, state-level health surveillance system generalizable to the Florida population to examine trends in PSA screening over time and across racial and ethnic groups. Another strength was our in-depth exploration of disparities in PSA screening frequency at the county level to better understand geographic heterogeneity in screening. Our study also has limitations. First, because BRFSS data are self-reported, the prevalence of prostate cancer screening may have been underestimated or overestimated, leading to measurement error, especially during the COVID-19 pandemic. Second, not all respondents eligible for the BRFSS prostate cancer screening module answered all the questions, resulting in missing data.
